# Mutational Landscape Assessed in Tumor Tissue and Circulating Tumor DNA During Treatment of Patients with HER2/*ERBB2*-Mutated Solid Tumors

**DOI:** 10.1186/s12885-025-14599-7

**Published:** 2025-08-06

**Authors:** Kristian Egebjerg, Iben Spanggaard, Lise Barlebo Ahlborn, Kristoffer Staal Rohrberg, Laurine Harsloef, Martin Hoejgaard, Ane Yde Schmidt, Ulrik Lassen, Ida Viller Tuxen, Jane Preuss Hasselby, Maria Rossing, Eric Santoni-Rugiu, Christina Westmose Yde, Morten Mau-Sørensen

**Affiliations:** 1 Department of Oncology, Copenhagen University Hospital, Rigshospitalet, 9, Blegdamsvej, Copenhagen, 2100 Denmark; 2 Center for Genomic Medicine, Copenhagen University Hospital, Rigshospitalet, Copenhagen, Denmark; 3 Department of Pathology, Copenhagen University Hospital, Rigshospitalet, Copenhagen, Denmark

**Keywords:** HER2, ERBB2, Mutations, ctDNA, Cancer

## Abstract

**Background:**

Human epidermal growth factor receptor 2 (HER2) aberrations, such as protein overexpression and amplification of the *HER2* gene (*ERBB2*), are well-established in breast and gastroesophageal adenocarcinomas. However, *ERBB2* oncogenic variants occur in 3.5% of all solid tumors with possible therapeutic implications. This study investigates the treatment efficacy and mutational landscape of patients with *ERBB2*-mutated cancers receiving HER2-targeted therapy.

**Methods:**

Nineteen patients with refractory solid tumors harboring *ERBB2* oncogenic variants were enrolled in the Copenhagen Prospective Personalized Oncology trial and received HER2-targeted treatment. Whole-exome sequencing, ctDNA analysis, and imaging were conducted at baseline, during treatment, and upon progression. Descriptive statistics were employed due to the exploratory nature of the study.

**Results:**

HER2-targeted treatment yielded a 37% overall response rate, a 68% disease control rate, and a median progression-free survival of 4.4 months. A tendency was observed toward higher overall response rate (60%) in patients harboring *ERBB2* oncogenic variants located in the tyrosine kinase domain. Clonality of *ERBB2* oncogenic variants was linked with treatment efficacy, underscoring the reduced effect when targeting subclonal mutations. Sequential ctDNA analysis of *ERBB2* oncogenic variants demonstrated correlation with treatment response.

**Conclusion:**

In this heterogeneous cohort of patients harboring *ERBB2* oncogenic variants, HER2-targeted therapy demonstrated clinical efficacy. Mutational analysis revealed the importance of clonal *ERBB2* oncogenic variants and identified factors influencing treatment outcomes. Limitations include a small sample size as well as heterogeneity in treatment regimens and cancer types.

**Supplementary Information:**

The online version contains supplementary material available at 10.1186/s12885-025-14599-7.

## Background

Human epidermal growth factor receptor 2 (HER2) is a tyrosine kinase of the epidermal growth factor receptor (EGFR) family encoded by a proto-oncogene, *ERBB2*, located on chromosome 17. The most prevalent aberrations of HER2 in cancer are HER2 protein overexpression and *ERBB2* gene amplification, present in 15–20% of breast and gastroesophageal adenocarcinomas [1]. HER2-targeted therapy is highly efficacious in this subset of patients [2, 3]. However, other aberrant variations of *ERBB2* exist in human cancer, including *ERBB2* oncogenic variants found in 3.5% of solid tumors, with varying prevalence across cancer types [4]. Several preclinical and clinical studies have reported promising efficacy of investigated HER2-targeted therapies in solid tumors harboring *ERBB2* oncogenic variants, potentially further expanding the population for which HER2-targeted treatment can be used [5–7]. Among these therapies, neratinib, a pan-HER tyrosine kinase inhibitor (TKI), has demonstrated promising clinical efficacy in both monotherapy- and combination regimens in patients with various solid tumors harboring *ERBB2* oncogenic variants, including breast, biliary tract, cervical, and non-small cell lung cancer [8–11]. Several factors are thought to influence the efficacy of HER2-targeted treatment directed at patients with *ERBB2-*mutated cancer, including cancer type, domain of the *ERBB2* oncogenic variant, as well as concurring co-mutations and other genomic alterations. Clonality of the *ERBB2* oncogenic variants and emergence of new driver mutations during treatment are potential explanations for treatment failure [12–14].

Clinical data and analysis on resistance in patients with *ERBB2* oncogenic variants treated with HER2-targeted therapy indicate that resistance predominantly arises through additional *ERBB2* oncogenic variants and/or amplification of the mutant allele, particularly in hormone-receptor positive metastatic breast cancer [8, 14]. In the SUMMIT and MutHER trials, dual HER2-targeting to address the increase in HER2 signaling was explored by adding trastuzumab to neratinib, with the anti-estrogen fulvestrant in HR-positive breast cancer in an effort to circumvent resistance [13–15]. As novel HER2-targeting regimens enter clinical practice and more patients with *ERBB2-*mutated tumors are eligible for targeted therapy, the need arises to further understand the evolution of the mutational landscape in the trajectory from diagnosis to refractory disease in different tumor types [13].

The aim of this study was to provide additional insights into the treatment efficacy and the dynamics of the tumor mutational landscape through sequential tissue biopsies and circulating tumor DNA (ctDNA) obtained at diagnosis, before, during, and after the HER2-directed treatment in patients with *ERBB2-*mutated solid tumors included in the Copenhagen Prospective Personalized Oncology (CoPPO– NCT02290522) trial at our institution [16].

## Materials and methods

### Patient cohort

Patients with refractory solid tumors were biopsied for whole-exome sequencing as part of the CoPPO program to identify actionable targets. Patients were enrolled from May 2013 to December 2021. Eligibility criteria included exhausted treatment options, a life expectancy of ≥ 3 months, normal organ function, Eastern Cooperative Oncology Group (ECOG) performance status (PS) of 0 or 1, age ≥ 18 years, and tumor lesions accessible for biopsy. The CoPPO program served as a framework to perform comprehensive molecular profiling locally and guide patients toward precision therapies or clinical trial enrollment. Patients with *ERBB2* oncogenic variants received HER2-targeted treatment either through the multicenter SUMMIT trial in HER2-mutant cancers, off-label use, or in a named patient program. The study was conducted in accordance with the Declaration of Helsinki and approved by an institutional review board and the Regional Ethics Committee (Danish Ethical Committee, file number: 1300530). All patients provided signed informed consent.

### Sample and data collection

Computed Tomography (CT) scans were performed every eight weeks, and tumor responses were evaluated according to RECIST 1.1 [17], classified as complete response (CR), partial response (PR), stable disease (SD), or progressive disease (PD).

When possible, archival formalin-fixed paraffin-embedded (FFPE) tissue from the primary tumor and prior metastases was collected. Fresh tumor biopsies were obtained at inclusion, during treatment and at progression, for comprehensive genomic analysis, if feasible. Additionally, plasma samples for ctDNA analysis were collected before, during treatment, and at progression. In total, 41 tumor samples were analyzed, including 12 FFPE biopsies and 29 biopsies preserved in RNA-later, along with 128 plasma ctDNA samples.

### Endpoints

Clinical endpoints included objective response (OR) and overall response rate (ORR) defined as CR and PR, and disease control rate (DCR) defined as CR, PR and SD. Further time until progression, (Progression Free Survival (PFS)) and time until death (Overall Survival (OS)) were recorded. Analysis of PFS2/PFS1 ratio was conducted [18]. PFS2 is defined as the PFS time associated with the *ERBB2* targeted therapy, and PFS1 the PFS time of the last prior systemic therapy. Follow-up was updated on March 23, 2023. Next, clinical endpoints were compared with data from translational molecular analysis to assess the following: (1) The impact of tumor mutations detected in solid and liquid biopsies before, during, and after HER2-targeted therapy on outcomes. (2) The utility of repeat ctDNA variant allele frequency (VAF) measurement of the *ERBB2* oncogenic variants during therapy in the detection of response to treatment and at the progression of disease.

### Molecular analysis– Tumor sequencing

Tumor tissue was analyzed to identify oncogenic variants and amplifications using methods as previously described [16].

### Molecular analysis– ctDNA sampling and sequencing

Collection of blood and extraction of cell free DNA (cfDNA) was conducted as previously described [19]. DNA libraries were prepared from a minimum of 10 ng cfDNA and hybridized using the TruSight Oncology (TSO) 500 HT gene panel (Illumina) or the NebNext Ultra kit (New England Biolabs) where sequence capture was performed using the double capture protocol from Nimblegen with a custom designed sequence capture library. Libraries were sequenced on Illumina platforms (NovaSeq6000 for TSO500 libraries and miSeq for custom gene panels) to a minimum median coverage of 600×. For samples with coverage below 600× relevant tumor variants were manually inspected. Platform selection was based on available sample quantity, timing of inclusion, and protocol development over the study period. Illumina TSO500 sequencing was performed on cfDNA in all patients except for patient-2, who was analyzed usinga custom panel prior to the implementation of TSO500, and patient-16 for whom a droplet digital PCR (ddPCR, BioRad) approach was utilized according to manufacturer’s instructions. All platforms provided adequate coverage.

Sequencing reads were processed using GATK Mutect2 Best Practices guidelines. Further filtering and inspection of oncogenic variants was performed using Clinical Insight (QCI) Interpret Translational software both from Qiagen. Somatic variants were classified according to Horak et al. based on recommendations of Clinical Genome Resource (ClinGen), Cancer Genomics Consortium (CGC), and Variant Interpretation for Cancer Consortium (VICC) [20].

Specifically, variants were categorized as ‘pathogenic/likely pathogenic’ (oncogenic drivers), ‘variants of unknown significance’ (VUS), or ‘benign/likely benign’ if they were known polymorphisms. Pathogenic or likely pathogenic somatic mutations including nonsense, frameshift, missense, and splice site alterations (+/−2 bp) in cancer-related genes were reported.

### Treatments

Eligible patients (*n* = 15) were enrolled in the SUMMIT study (NCT01953926). Breast cancer patients were treated with neratinib monotherapy, neratinib + fulvestrant, neratinib + trastuzumab, or the triplet combination of neratinib + fulvestrant + trastuzumab, depending on hormone receptor status and the study phase at the time of inclusion [7]. Patients with urothelial carcinoma were treated with a combination of neratinib and paclitaxel. Dosing was administered according to the prior publications [7, 8, 14] Patients not participating in the SUMMIT trial (*n* = 4) received different HER2-directed therapies: Epirubicin (90 mg/m^2^ IV Q3W) + trastuzumab; trastuzumab-emtansine (T-DM1) (3.6 mg/kg Q3W) or neratinib monotherapy (in a named patient program).

### Statistical considerations

Due to the number of patients included and the exploratory nature of the study, results are reported descriptively. 95% Confidence Intervals (95% CI) were calculated using t-test for numerical data and binomial exact calculation for proportions respectively. The log-rank test was used to compare PFS between treatment groups (monotherapy vs. combination therapy), cancer types, and the genomic location of oncogenic variants, while the Wilcoxon rank-sum test was applied to compare PFS2/PFS1 ratios across the same groups. Fisher’s exact test was utilized to calculate *p*-value for the association of ctDNA and best radiological response.

### Previous data on select patients

For nine patients, data on response and baseline mutations have been previously published as part of the SUMMIT trial, with serial ctDNA analysis reported for one of these patients [7–10, 14]. However, this study provides novel insights for all nine patients, specifically regarding PFS2/1, ctDNA, and sequential mutational analyses of tumor tissue during HER2 directed treatment. Please refer to the supplementary Table 1 for complete details.

## Results

### Patient cohort

**Fig. 1:**
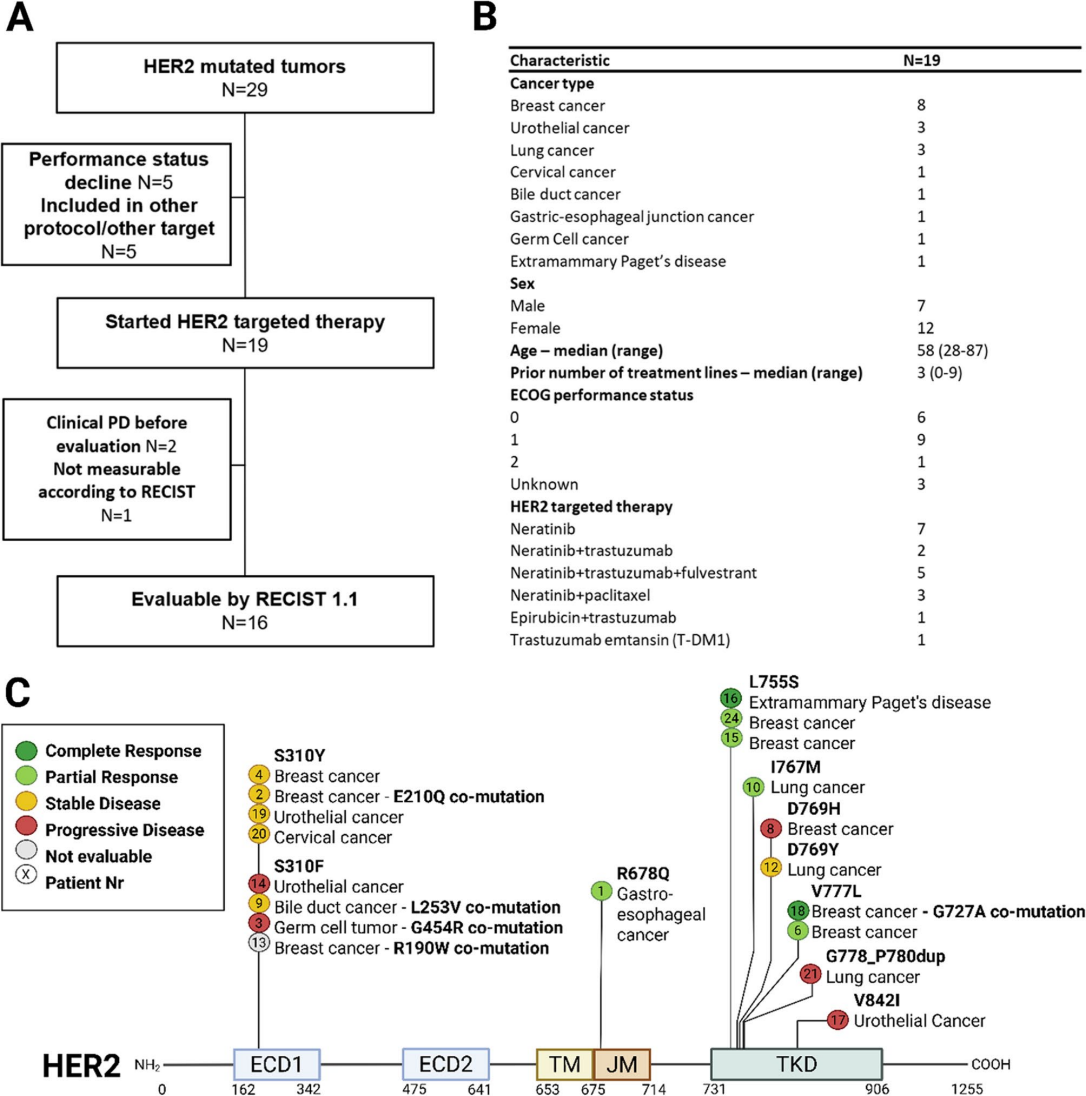
**A** Consort diagram showing the flow of patients selected for HER2-directed therapy in the CoPPO study **B** Characteristics of patients with HER2/*ERBB2-*mutated tumors, including tumor type and assigned therapy. **C** Lollipop mutation diagram of the HER2-protein, indicating the location and type of mutations for each specific patient included. ECD: Extracellular Domain, TM: Transmembrane Domain, JM: Juxtramembrane Domain, KD: Kinase Domain

From February 2013 to December 31, 2021, twenty-nine patients with stage IV disease harboring somatic *ERBB2* oncogenic variants were identified through the CoPPO Program of whom nineteen commenced HER2-targeted therapy including breast cancer (*n* = 8), urothelial carcinoma (*n* = 3), non-small cell lung cancer (*n* = 3), and one case each of cervical cancer, cholangiocarcinoma, gastroesophageal junction cancer, germ cell tumor, and extramammary Paget’s disease (Fig. 1). At baseline, two patients were also HER2-positive according to standard immunohistochemistry/in situ hybridization and one patient had *ERBB2* gene amplification, two patients had received prior HER2-targeted therapy. Seven out of eight breast cancer (BC) patients were estrogen receptor (ER) positive (Full data and characteristics can be found in Supplemental Table 2). A total of six different HER2-targeted regimens were utilized. Fifteen patients were included in the SUMMIT trial, and four patients received HER2-targeted treatment either off-label or in a named-patient program.

### Treatment efficacy 

**Fig. 2:**
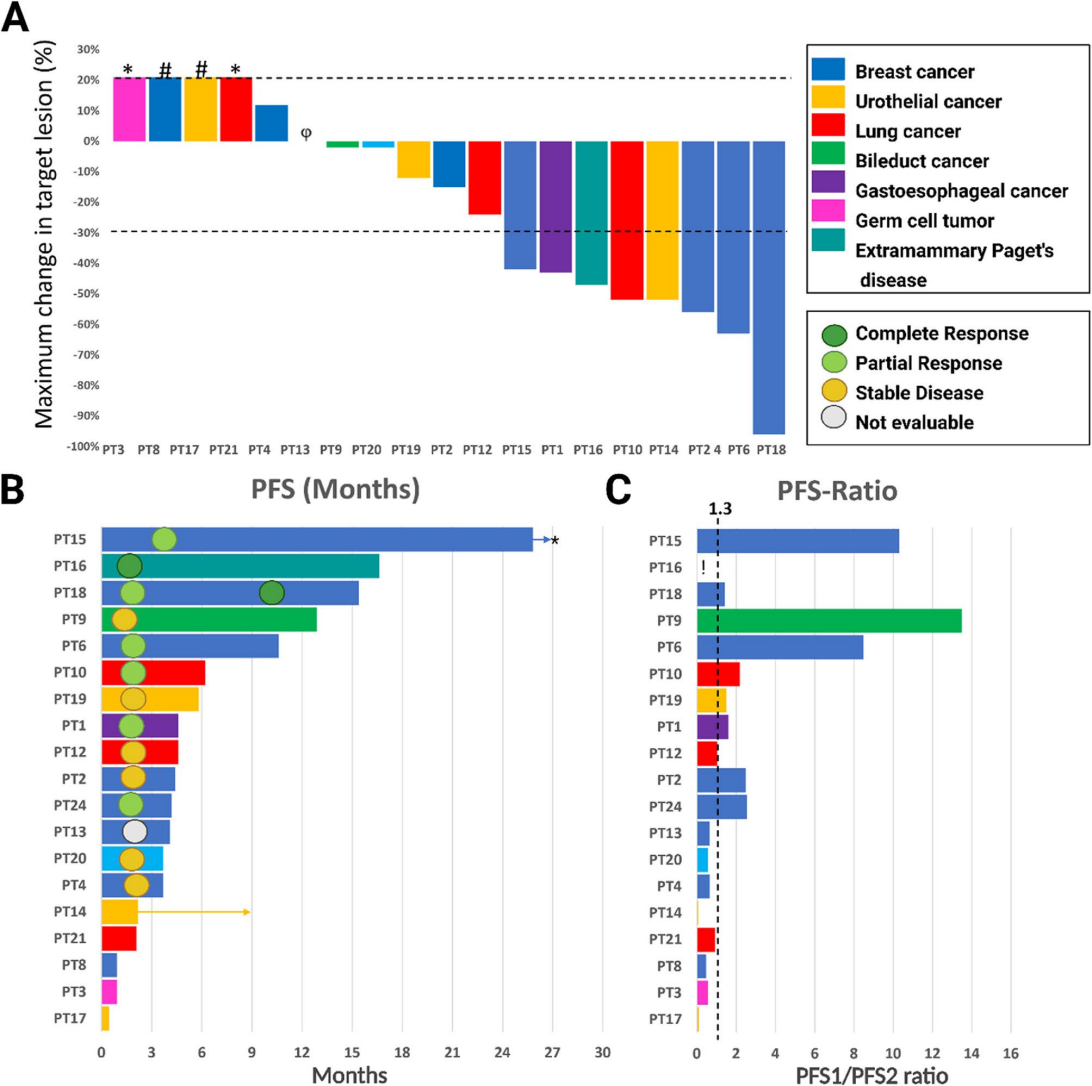
**A** Waterfall plot illustrating the maximum change in the sum of target tumor lesions from baseline to the best response, following RECIST 1.1 criteria. Increments above 20% indicate progressive disease, reductions greater than 30% indicate a response, and changes between 20% and -30% indicate stable disease. Each patient is represented by a colored bar corresponding to their cancer type, as indicated in the legend. Patients experiencing progression due to new lesions, unequivocal worsening of non-measurable disease, death from the disease, symptomatic deterioration, or inadequate assessment (considered failures as per outcome definitions in each protocol) are denoted by bars showing a 21% increase in tumor lesions. † PD due to death, *PD due to new lesion, #PD due to clinical PD, φ Not evaluable according to RECIST. **B** Swimmer plot depicting disease progression assessed by RECIST 1.1, represented by a colored horizontal line for each patient. The time and type of the best response according to RECIST 1.1 are marked on the axis by a circle. An arrow indicates treatment beyond progression for patients 14 and 15. *Patient 15 is still undergoing treatment beyond progression, currently at 38 months of treatment duration. **C** Plot of PFS2/PFS1 ratio. PFS2 refers to the PFS of the HER2 directed treatment in this study, and PFS1 refers to the PFS of the most recent treatment before inclusion in this study. A dashed line marks the PFS2/PFS1 ratio of 1.3. PT16 did not receive prior treatment before inclusion in this study

Out of the nineteen patients who received treatment, eighteen were evaluable according to RECIST 1.1 criteria. Two patients experienced clinical PD before initial evaluation, and one patient did not have measurable disease and was not evaluable according to RECIST 1.1 (Fig. 2). Treatment was ongoing for one patient at data cut off. OR was observed in seven patients (37%, 95% CI: 16–62%), with two patients achieving CR and five patients achieving a PR. Six patients had SD, resulting in a DCR of 68%. Five patients had PD as best response. Median PFS was 4.4 months (95% CI: 3.6–10 months). Patient-15 experienced PD at 26 months due to the emergence of a solitary liver metastasis, which was surgically removed. This patient continues treatment beyond progression and has at the time of data cut off received treatment for 38 months. Out of the nineteen patients who received treatment, eighteen had received prior treatment. Of these, nine patients (50%, 95% CI 26–74%) had a PFS2/PFS1 ratio of > 1.3. Furthermore, three patients exhibited markedly high PFS2/PFS1 ratios: patient-15 = 10.3, patient-9 = 13.5, and patient-6 = 8.5. Patient-16 had not received any prior systemic treatment, as there are currently no well-established first-line therapies for patients with disseminated extramammary Paget’s disease. We evaluated outcomes by oncogenic variant domain, tumor type, and treatment regimen. Patients with extracellular domain (ECD) oncogenic variants had a median PFS of 3.9 months and a median PFS2/PFS1 ratio of 0.66, while those with kinase domain (KD) oncogenic variants had a median PFS of 5.4 months and a ratio of 1.24. By cancer type, breast cancer patients showed a median PFS of 4.3 months and a PFS ratio of 1.97. For NSCLC, median PFS was 4.6 months and the ratio 1.04. In urothelial cancer, PFS was shorter at 2.2 months with a PFS ratio of 0.11. Patients receiving combination HER2-targeted therapy (*n* = 11) had a median PFS of 4.6 months and a PFS ratio of 1.50, compared to 3.7 months and 0.62, respectively, in those on monotherapy (*n* = 7). None of these differences were statistically significant.

### Baseline *ERBB2*- and co-mutations

**Fig. 3:**
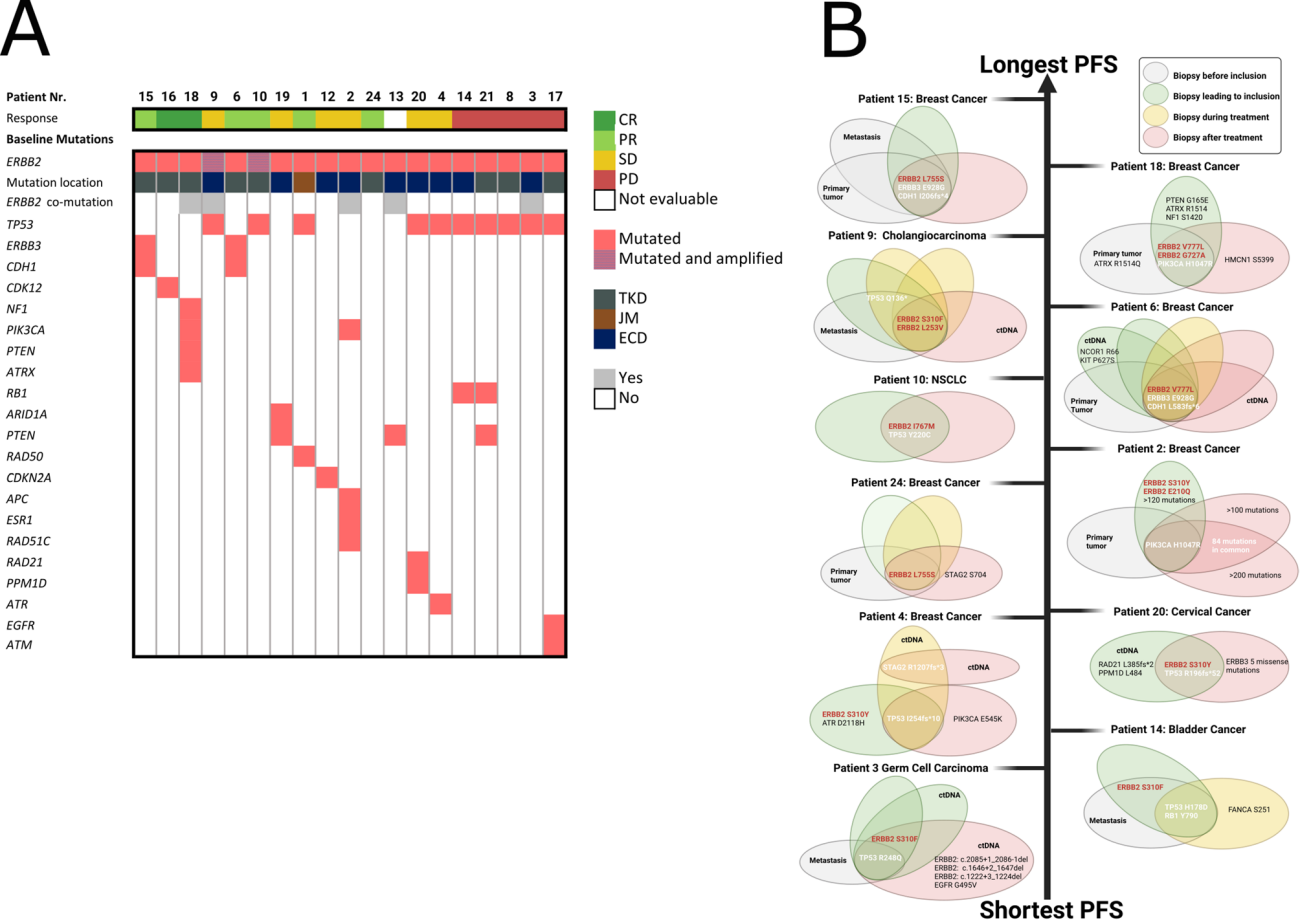
**A** Baseline co-occurring genomic alterations with HER2/*ERBB2*-oncogenic variants. Responses according to RECIST are color-coded as follows: dark green = Complete Response, light green = Partial Response, yellow = Stable Disease, red = Progression of Disease, white = Not Evaluable according to RECIST. The location of the *ERBB2* oncogenic variants is color-coded as follows: dark green = Tyrosine Kinase Domain, brown = Juxtamembrane region, dark blue = Extracellular Domain. Patients are listed from left to right based on duration of PFS. **B** Venn diagrams illustrating patients with multiple biopsies and identified mutations. Patients are listed on a y-axis from longest to shortest PFS. The color of the Venn diagram indicates the time of biopsy: grey indicates mutational analysis from a biopsy taken before inclusion, green from a biopsy leading to inclusion, yellow from a biopsy during treatment, and red from a biopsy after treatment. Red text marks the *ERBB2* oncogenic variant. White text indicates mutations located in several biopsies in overlapping Venn diagrams

Twelve patients had somatic *ERBB2* oncogenic variants in the ECD, including five with S310Y and seven with S310F (Table 1) (Fig. 1C). One patient had an R678Q oncogenic variants in the juxtamembrane domain (JMD), and ten patients had oncogenic variants in the KD. Eight of the patients with KD oncogenic variants were clustered around residues 755–780. The most prevalent oncogenic variants were S310F, S310Y, and L755S. Five patients had additional oncogenic variants in *ERBB2*, with all five co-mutations located in the same domain as the primary oncogenic variant. The two patients with the longest PFS harbored L755S mutations. Six out of ten patients with* ERBB2* oncogenic variants in the KD had an OR. Notably, both patient-6 and patient-15 had long-lasting clinical responses and a similar baseline mutation pattern: the *ERBB2* oncogenic variant was located in the KD, and both had baseline *ERBB3* and *CDH1* co-mutations (Fig. 3a).

**Table 1:** Characteristics of patients included in the study. PS (ECOG performance Status)

Patient ID	Cancer type	Gender	Age	PS	Baseline *ERBB2* oncogenic variant	Baseline *ERBB2* co-mutations	Treatment
1	Gastro-esophageal junction: Adenocarcinoma	Male	51	*N/A*	R678Q		Epirubicin + trastuzumab
2	Breast cancer: Invasive Ductal Carcinoma	Female	56	*N/A*	S310Y	E210Q	Neratinib
3	Germ Cell Cancer: Choriocarcinoma	Female	28	*N/A*	S310F	G454R	Neratinib
4	Breast cancer: Invasive Ductal Carcinoma	Female	87	1	S310Y		Neratinib
6	Breast cancer: Invasive Lobular Carcinoma	Female	38	0	V777L		Neratinib + trastuzumab + fulvestrant
8	Breast cancer: Invasive Ductal Carcinoma	Female	52	1	D769H		Neratinib
9	Biliary Tract Cancer: Cholangiocarcinoma	Male	50	1	S310F	L253V	Neratinib
10	NSCLC: Adenocarcinoma	Female	68	0	I767M		Neratinib + trastuzumab
12	NSCLC: Adenocarcinoma	Male	69	1	D769Y		Neratinib + trastuzumab
13	Breast cancer: Invasive Lobular Carcinoma	Female	58	0	S310F	R190W	Neratinib + trastuzumab + fulvestrant
14	Bladder cancer: Urothelial carcinoma:	Male	62	1	S310F		Neratinib + paclitaxel
15	Breast cancer: Invasive Lobular Carcinoma	Female	49	0	L755S		Neratinib + trastuzumab + fulvestrant
16	Extramammary Paget’s disease: Scrotum	Male	65	0	L755S		Neratinib
17	Renal Pelvis Cancer: Urothelial carcinoma	Female	73	2	V842I		Neratinib + paclitaxel
18	Breast cancer: Invasive Ductal Carcinoma	Female	58	1	V777L	G727A	Neratinib + trastuzumab + fulvestrant
19	Bladder cancer: Urothelial carcinoma	Male	79	1	S310Y		Neratinib + paclitaxel
20	Cervical cancer: Squamous Cell carcinoma	Female	72	1	S310Y		Neratinib
21	NSCLC: Adenocarcinoma	Male	46	1	G778_P780dup		Trastuzumab emtansin (T-DM1)
24	Breast cancer: Invasive Ductal Carcinoma	Female	47	0	L755S		Neratinib + trastuzumab + fulvestrant

### Detection of activating *ERBB2* oncogenic variants throughout the disease course

Tumor tissue from prior biopsies was successfully collected and analyzed in eight patients (Fig. 3b). The *ERBB2* oncogenic variant was identified in archival tissue for six of these patients. Notably, four of these patients achieved the longest PFS, and in patient-14, the oncogenic variant was detected in a prior metastasis. In the two patients where the *ERBB2* oncogenic variant was not detectable, the PFS was 4 and 19 weeks, respectively. For three out of eleven patients with mutational analysis of repeated biopsies during or following HER2-directed therapy, the *ERBB2* oncogenic variant was not detectable. Among these, two patients experienced progressive disease shortly after this biopsy. One patient continued treatment for six more months after the *ERBB2* oncogenic variant was not detected before eventually experiencing progressive disease. Among the four patients in whom the *ERBB2* oncogenic variant was either not detectable in prior tissue or not continuously detectable during treatment, the mean PFS was 2.8 months (range: 0.9–4.4 months). For the six patients in whom the *ERBB2* oncogenic variant was detectable in archival tissue mean PFS was 12 months (range: 2.2–26 months).

### Emerging driver mutations

Eleven patients underwent either liquid (*n* = 5) or solid tumor biopsies (*n* = 14) for the analysis of the mutational landscape during or after treatment. Among them, additional driver mutations were identified (Fig. 3a-b). Four patients exhibited new mutations in the HER family, involving *EGFR*, *ERBB2*, *ERBB3*, or downstream signaling through *PIK3CA*, potentially explaining resistance to treatment.

### Longitudinal ctDNA measurements

**Fig. 4:**
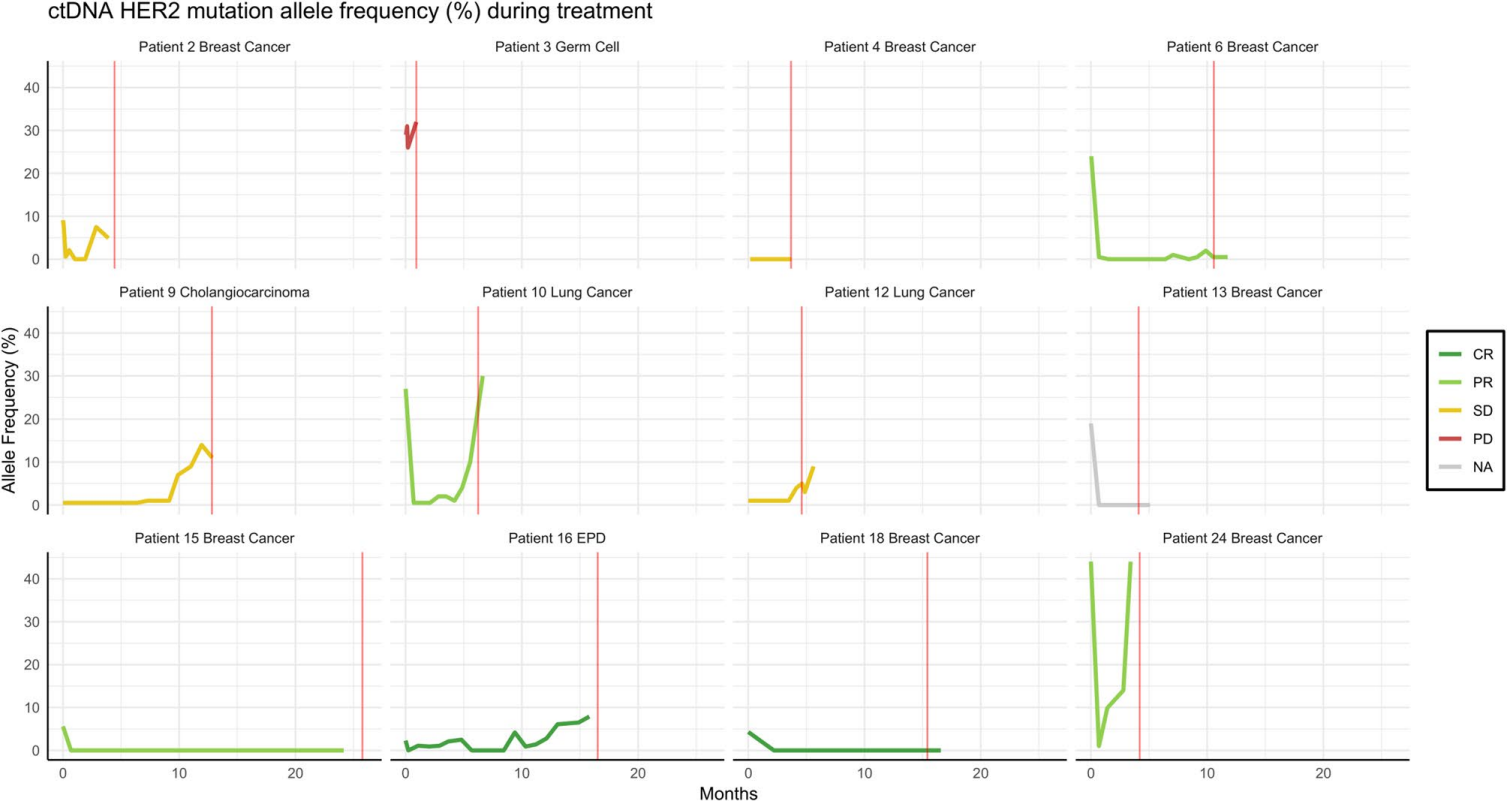
Plot of patients with continuous measurement of ctDNA during treatment. Each patient is labeled according to the legend. The X-axis represents time in months from the start of therapy. The Y-axis indicates the allele frequency in plasma as a percentage for the specific HER2/*ERBB2*-oncogenic variant. The vertical red line denotes the time of clinical or radiological progression of the disease. The lines are color coded based on best response according to RECIST. EPD = Extramammary Paget’s Disease, CR=Complete Response, PR= Partial Response, SD=Stable Disease, PD=Progressive Disease, NA=Not Available for evaluation according to RECIST

Fourteen patients underwent ctDNA analysis to assess the allele frequency of their specific *ERBB2* oncogenic variant, which was detectable in thirteen patients. Sequential ctDNA analysis during and after treatment was possible for twelve patients (Fig. 4; see Supplemental Figures for individual timelines). There was a statistically significant (*p* = 0.01) association between ctDNA change and best response (Supplementary Fig. 1): After starting treatment, three patients exhibited unchanged levels of ctDNA and one patient a slight increase in ctDNA. Patients with unchanged levels had SD at radiological evaluation while one was not evaluable, while the patient with a slight increase in ctDNA had PD. Eight patients showed a decrease in ctDNA allele frequency by more than 50%. At radiological evaluation of these eight patients, two had CR, five PR, and one SD per RECIST 1.1, respectively. In seven patients, an increase in ctDNA by more than 50% preceded the radiological detection of progressive disease at the subsequent tumor evaluation.

## Discussion

Mutational analysis before, during, and after treatment revealed improved efficacy in patients in whom the *ERBB2* oncogenic variants were detectable throughout the course of the disease, from the time of diagnosis to the development of treatment-refractory metastases. This confirms the limited efficacy of targeting subclonal *ERBB2* oncogenic variants [14]. Sub-clonal *ERBB2* mutations have been shown to be more prevalent in patients with *ERBB2* amplified tumors compared to HER2-negative tumors [21]. Clonal mutations represent robust therapeutic targets, whereas subclonal mutations—typically emerging under therapy—may signal non-dominant passenger mutations or acquired resistance. Detecting these subclonal mutations can serve as an early warning of treatment failure, prompting consideration of alternative therapeutic strategies. Moreover, in patients where the *ERBB2* oncogenic variant was not detectable in prior tissue biopsies, this may reflect subclonality or spatial heterogeneity, raising concerns about the potential effectiveness of HER2-targeted treatment. For such patients, earlier consideration of combination regimens or alternative treatment approaches may be warranted. The patients in our cohort have undergone extensive pre-treatment for advanced disease, and as such, some degree of sub-clonality is to be expected. Eight patients had ECD *ERBB2* oncogenic variants (S310F/Y). The most common *ERBB2* oncogenic variant in bladder cancer and one of the most common *ERBB2* oncogenic variants overall. The missense S310F/Y oncogenic variants cause changes in the furin-like receptor coding for the ECD. Domain II/CR1 of HER2 thereby generates structural changes promoting dimerization and kinase activation [22, 23]. As domain II/CR1 is affected, pertuzumab reactivity is thought to be abolished due to the localization of its binding site on II/CR1. In contrast, S310 oncogenic variants have been correlated with trastuzumab and tyrosine kinase inhibitors (TKI) sensitivity in preclinical models [23]. It has been argued that in patients harboring S310 oncogenic variants, additional *ERBB2* co-mutations can cause drug resistance [22]. Four patients with primary S310F/Y oncogenic variants had a baseline *ERBB2* co-mutation.

Only in four out of eleven patients did new mutations occur in the HER-family of genes or in the genes coding for the downstream signaling pathway of HER2. In total, ten patients had a baseline TP53 mutation and in one patient an emergent TP53 mutation appeared. In a cross-trial analysis of ERBB2-mutated patients, TP53 mutations exhibited a trend toward a worse prognosis with anti-HER2 TKI treatment than TP53‐wild‐type patients [24]. Further, Shishido et al. conducted sequential mutational analysis of five ERBB2-mutated breast cancer patients treated with neratinib and also showed new mutations arising in ERBB2, PIK3CA, and TP53 in a patient with treatment failure [25] In this cohort HER2-targeted treatment resulted in an overall response rate of 37%, a disease control rate of 68%, and a median progression-free survival (PFS) of 4.4 months. Our response rate was similar to that of another basket trial in the same clinical setting of *ERBB2*-mutated patients, where the overall response rate was observed in 36% of patients [26]. The DCR and ORR in our cohort are numerically higher compared to other studies investigating neratinib in non-breast cancer patients [9–11]. This may be partially explained by the inclusion of breast cancer patients in our cohort. In a publication by Jhaveri et al., the ORR among patients with metastatic HER2-mutant, ER+/HER2- breast cancer treated with neratinib, fulvestrant, and trastuzumab was 39% [8]. In our study, among the patients who received monotherapy with neratinib, only one out of seven achieved OR to treatment, while six out of eight patients with neratinib combination regimens and evaluable disease demonstrated an OR.

The utilization of ctDNA for monitoring of the targeted mutation can potentially be a useful supplementary tool for monitoring response and resistance to treatment, allowing for the early cessation of futile therapy. ctDNA monitoring demonstrated a statistical association with radiological response. Concerning the progression of the disease, an increase in ctDNA nearly always indicated disease progression, although not all patients exhibited an increase in ctDNA before progression. These findings closely align with the results of *Ma* et al., who investigated cfDNA in eleven *ERBB2*-mutated breast cancer patients receiving neratinib [27].

As not all patients had any identifiable new driver mutations, this highlights that treatment failure cannot be explained solely based on new mutations. Other factors, such as copy-number variations, changes in the transcriptome, proteome, and tumor milieu, should be explored further. Our study provides new insights into the mutational landscape in a field with very limited data. Our study has a limited number of patients, and the generalizability of our results is limited. The rarity of *ERBB2* oncogenic variants and the limited accessibility to molecular profiling and HER2-targeted therapies pose challenges in recruiting larger cohorts. The treated population is very heterogeneous with seven different treatment regimens utilized on six different cancer types. Our study is also limited, as we could only access archival tumor tissue in a limited number of patients. Further sequential acquisition of tumor tissue and ctDNA was not possible in all patients.

In conclusion, in our cohort of patients with various *ERBB2-*mutated tumor types who had exhausted standard treatment options, HER2-targeted treatment resulted in clinically meaningful efficacy in a substantial number of patients. Sequential mutational analysis highlighted the importance of identifying and treating patients harboring *ERBB2* oncogenic variants originating from the primary tumor. Data indicate that clinical benefit seems larger in patients with oncogenic variants in *ERRB2* kinase domain compared to *ERBB2* extracellular domain. The presence of co-mutations and acquisition of new driver mutations seemed related to short duration of benefit and treatment failure.

## Supplementary Information

Supplementary Material 1.

## Data Availability

Given the small study population, the decision to share the patient level data needs to be handled on a case-by-case basis to determine if the clinical data can be adequately anonymized to give an acceptably low risk of patient identification. Requests should be sent to the corresponding author.

## Abbreviations

cfDNA: Cell-free DNA
CI: Confidence interval
CR: Complete response
CR1: Cysteine-rich domain 1
ctDNA: Circulating tumor DNA
DCR: Disease control rate
ECD: Extracellular domain
*EGFR*: Epidermal growth factor receptor
*ERBB2*: Human epidermal growth factor receptor 2 gene
*ERBB3*: Erb-B2 receptor tyrosine kinase 3
HER family: Human epidermal growth factor receptor family
HER2: Human epidermal growth factor receptor 2
KD: Kinase domain
OR: Objective response
ORR: Overall response rate
PD: Progressive disease
PFS: Progression-free survival
PFS1: Progression-free survival associated with the last prior systemic therapy
PFS2: Progression-free survival associated with HER2-targeted therapy
PR: Partial response
RECIST: Response evaluation criteria in solid tumors
SD: Stable disease
TKI: Tyrosine kinase inhibitor
