# Supplementary material for: Mutational Landscape Assessed in Tumor Tissue and Circulating Tumor DNA During Treatment of Patients with HER2/ERBB2-Mutated Solid Tumors
Source: BMC Cancer. 2025 Aug 6;25:1272. doi: 10.1186/s12885-025-14599-7 (PMC12330086; doi:10.1186/s12885-025-14599-7)

# **Supplement Online Content: Mutational landscape assessed in tumor tissue and circulating tumor DNA during treatment of patients with HER2/ERBB2-mutated solid tumors**

# **Supplemental Table 1: Previously published data**

| Patient ID | Prior reported data | Publication | Novel data |
| --- | --- | --- | --- |
| 1 | No |  |  |
| 2 | No |  |  |
| 3 | No |  |  |
| 4 | Baseline mutation and clinical response | Smyth et al 2020 | Longitudinal Mutations, ctDNA, PFS2/1 |
| 6 | Baseline mutation and clinical response | Jhaveri et al 2023 | Longitudinal Mutations, ctDNA, PFS2/1 |
| 8 | Baseline mutation and clinical response | Hyman et al 2018 | PFS2/1 |
| 9 | Baseline mutation and clinical response | Harding et al 2023 | Longitudinal Mutations, ctDNA, PFS2/1 |
| 10 | No |  |  |
| 12 | No |  |  |
| 13 | Baseline mutation and clinical response | Jhaveri et al 2023 | ctDNA, PFS2/1 |
| 14 | No |  |  |
| 15 | Baseline mutation and clinical response | Jhaveri et al 2023 | Longitudinal Mutations, ctDNA, PFS2/1 |
| 16 | No |  |  |
| 17 | No |  |  |
| 18 | Baseline mutation and clinical response | Jhaveri et al 2023 | Longitudinal Mutations, ctDNA, PFS2/1 |
| 19 | No |  |  |
| 20 | Baseline mutation and clinical response | Friedman et al 2024 | Longitudinal Mutations, PFS2/1 |
| 21 | No |  |  |
| 24 | Baseline mutation and clinical response, ctDNA | Jhaveri et al 2023 | Longitudinal mutations, ctDNA on own assay |

# **Supplemental Table 2: Characteristics of included patients**

| Patient ID | Protocol | HER2 status | Prior HER2 therapy | ER status | Detection *ERBB2* mutation in prior tissue | Continuous detection of *ERBB2* mutation |
| --- | --- | --- | --- | --- | --- | --- |
| 1 | Off - label | HER2pos (IHC) | Yes |  |  |  |
| 2 | Named patient programme | HER2neg | No | ERpos | No | No |
| 3 | Named patient programme |  | No |  | No | Yes |
| 4 | SUMMIT | HER2neg | No | ERpos |  | No |
| 6 | SUMMIT | HER2neg | No | ERpos | Yes | Yes |
| 8 | SUMMIT | HER2pos (IHC+ISH) | Yes | ERneg |  |  |
| 9 | SUMMIT |  | No |  | Yes | Yes |
| 10 | SUMMIT | *ERBB2*amp (SNP-Array) | No |  |  | Yes |
| 12 | SUMMIT |  | No |  |  |  |
| 13 | SUMMIT | HER2neg | No | ERpos |  |  |
| 14 | SUMMIT |  | No |  | Yes | No |
| 15 | SUMMIT | HER2neg | No | ERpos | Yes | Yes |
| 16 | SUMMIT | HER2neg | No |  |  |  |
| 17 | SUMMIT |  | No |  |  |  |
| 18 | SUMMIT | HER2neg | No | ERpos | Yes | Yes |
| 19 | SUMMIT |  | No |  |  |  |
| 20 | SUMMIT |  | No |  |  | Yes |
| 21 | Off - label |  | No |  |  |  |
| 24 | SUMMIT | HER2neg | No | ERpos | Yes | Yes |

# **Supplemental Figure 1: Association of initial ctDNA change and best objective response according to RECIST**


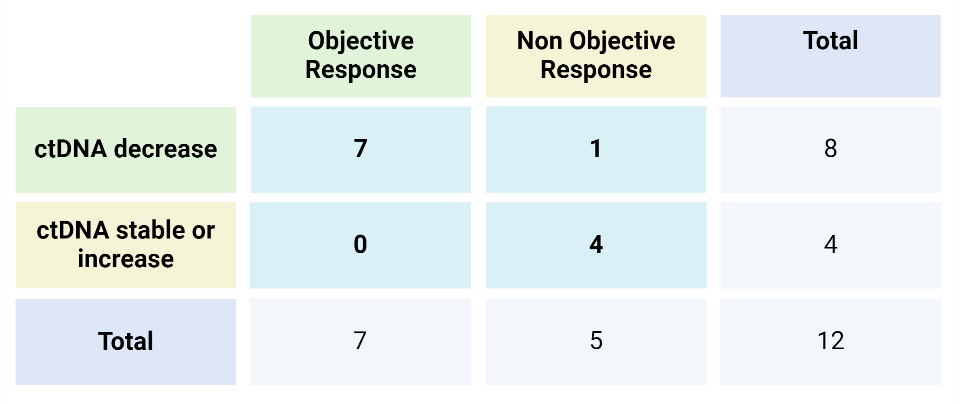


# **Supplemental Figures of ctDNA over the treatment course of HER2/*ERBB2* mutated patients**

Each figure represents the patient’s allele fraction of the respective HER2/*ERBB2* mutation detected from ctDNA over the course of treatment. Time point of collection of ctDNA is shown with a blue circle. Imaging from target lesions at evaluation at baseline and response are shown.


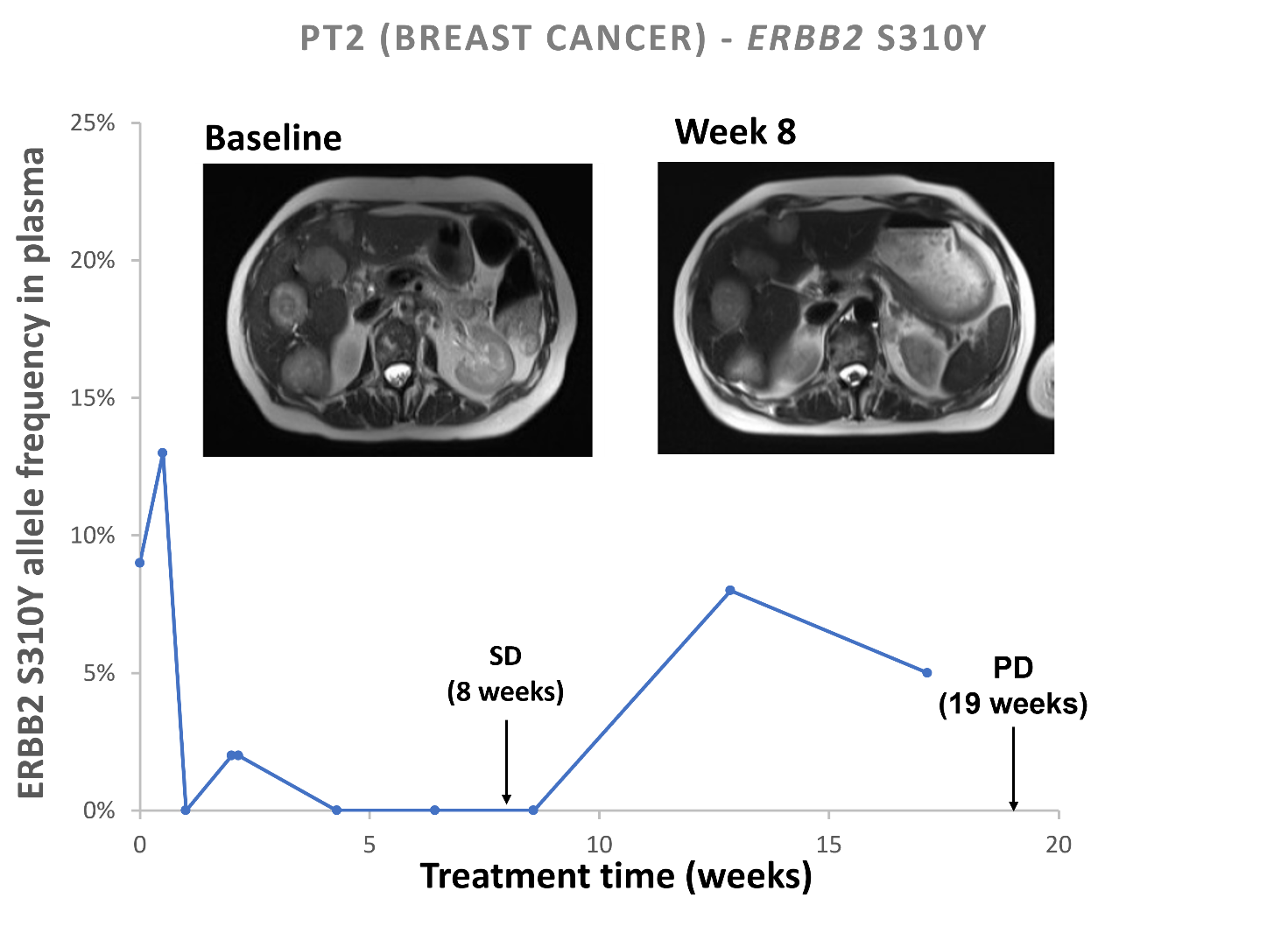


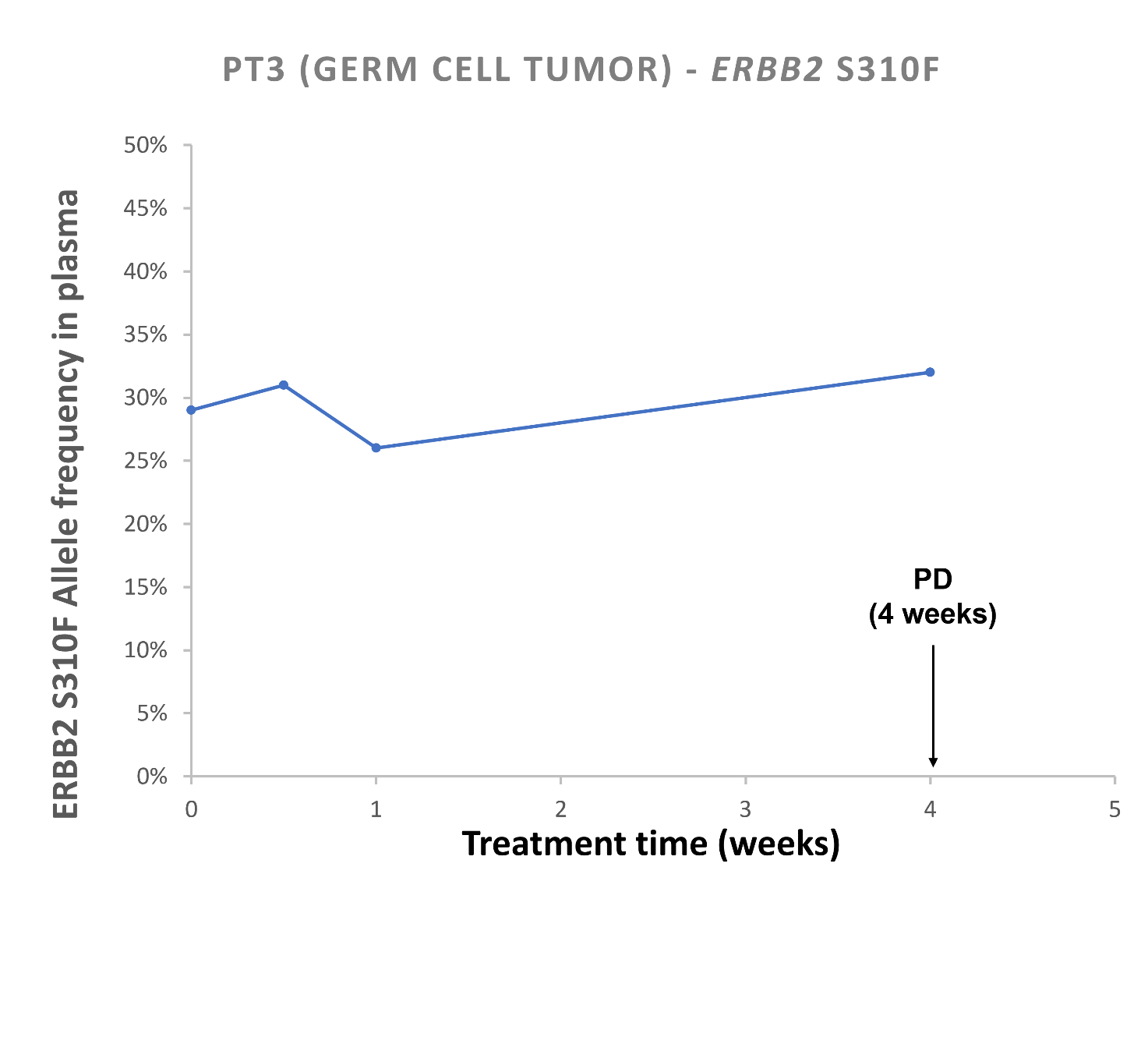


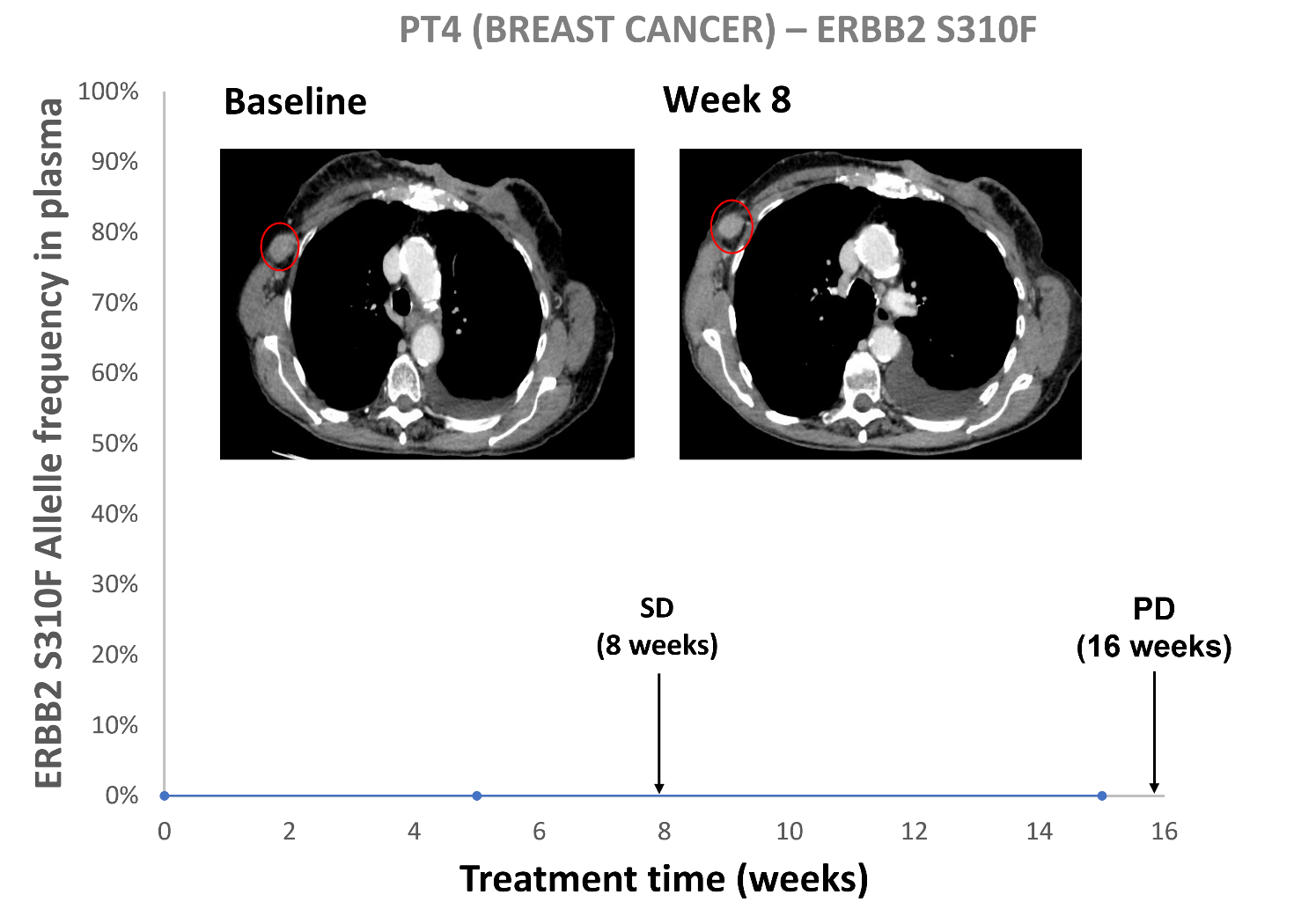


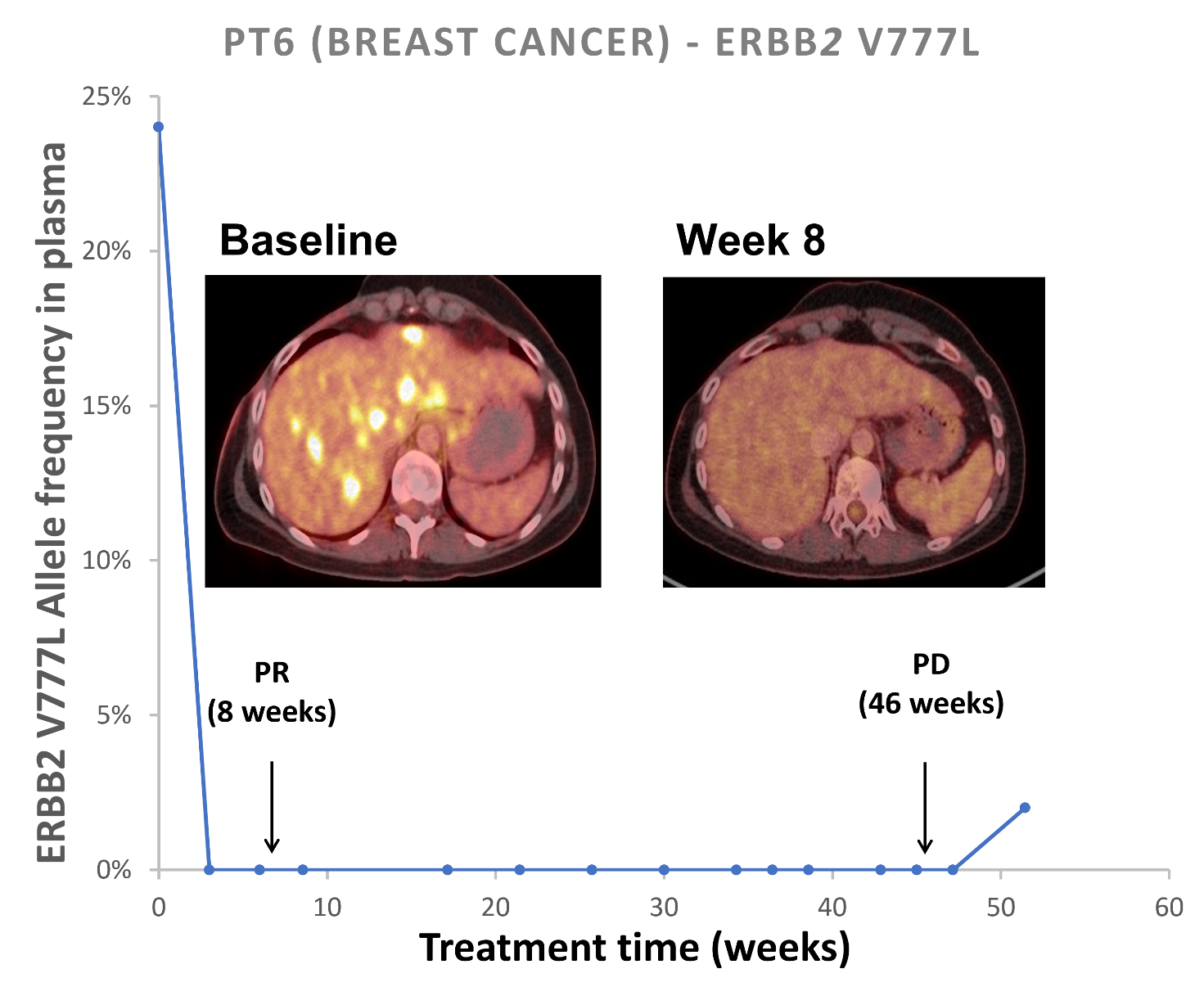


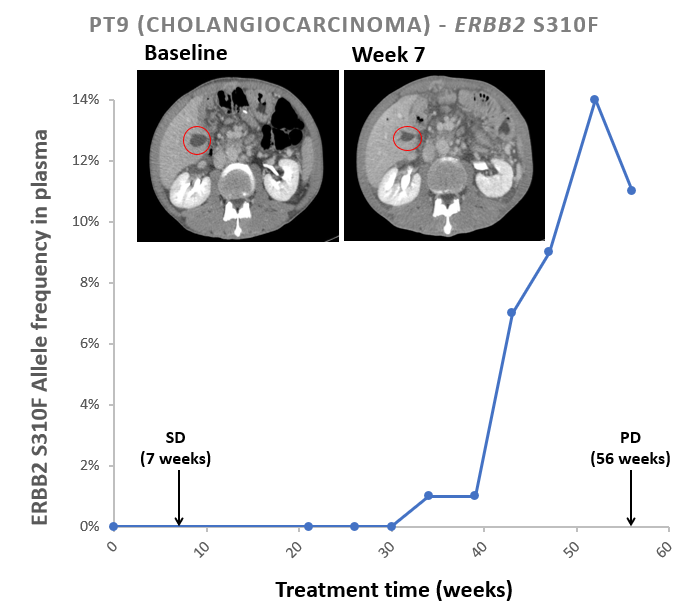


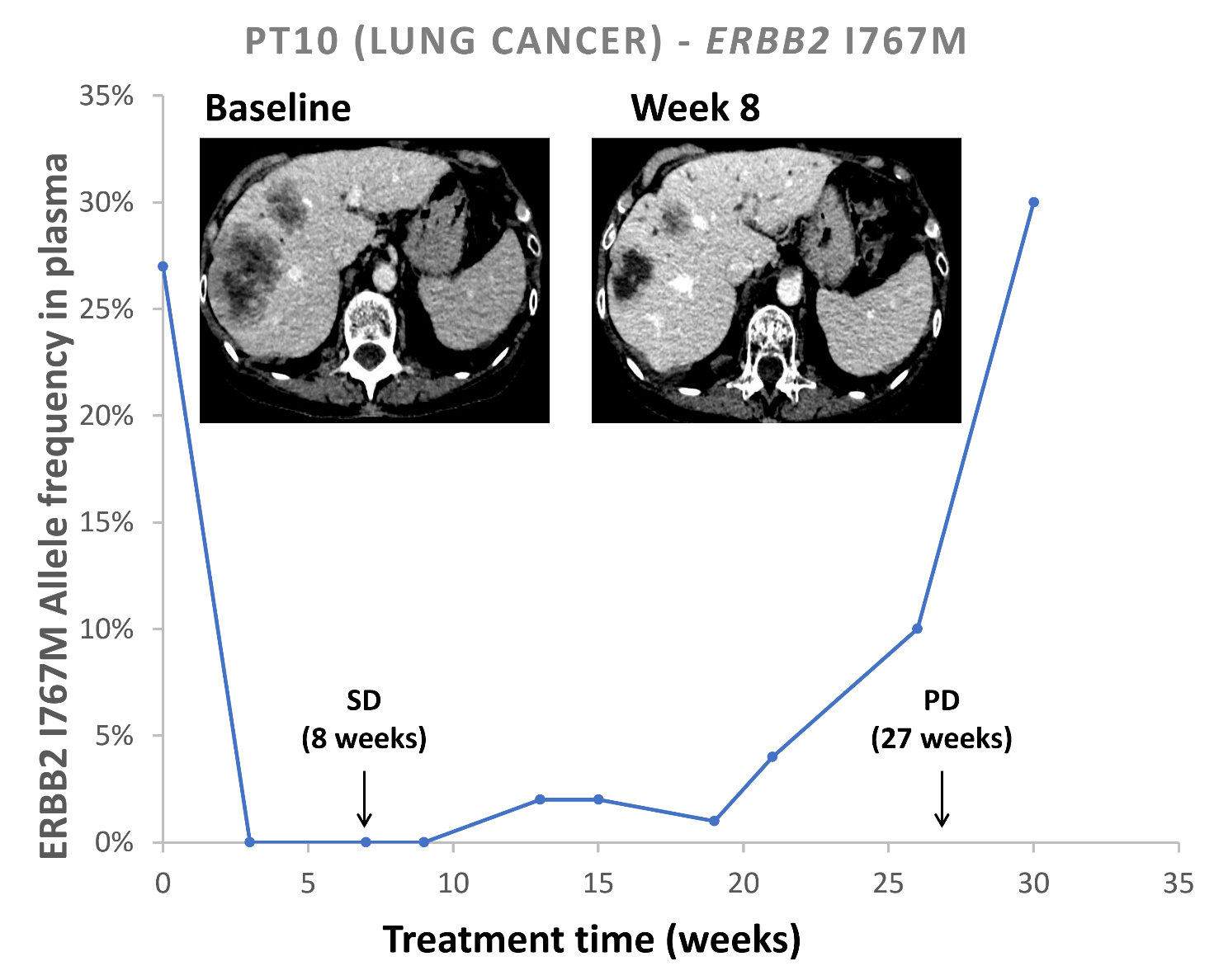

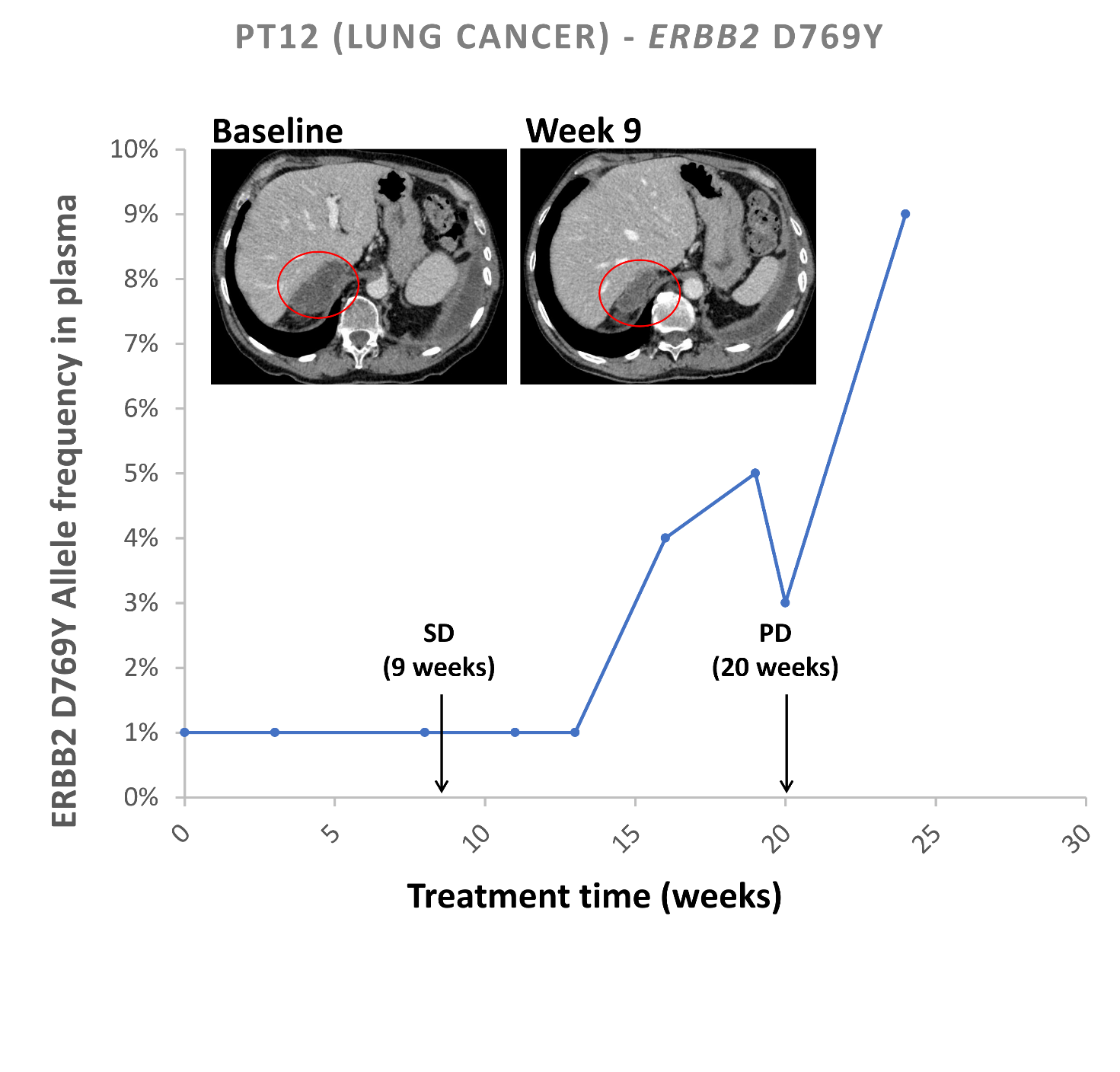

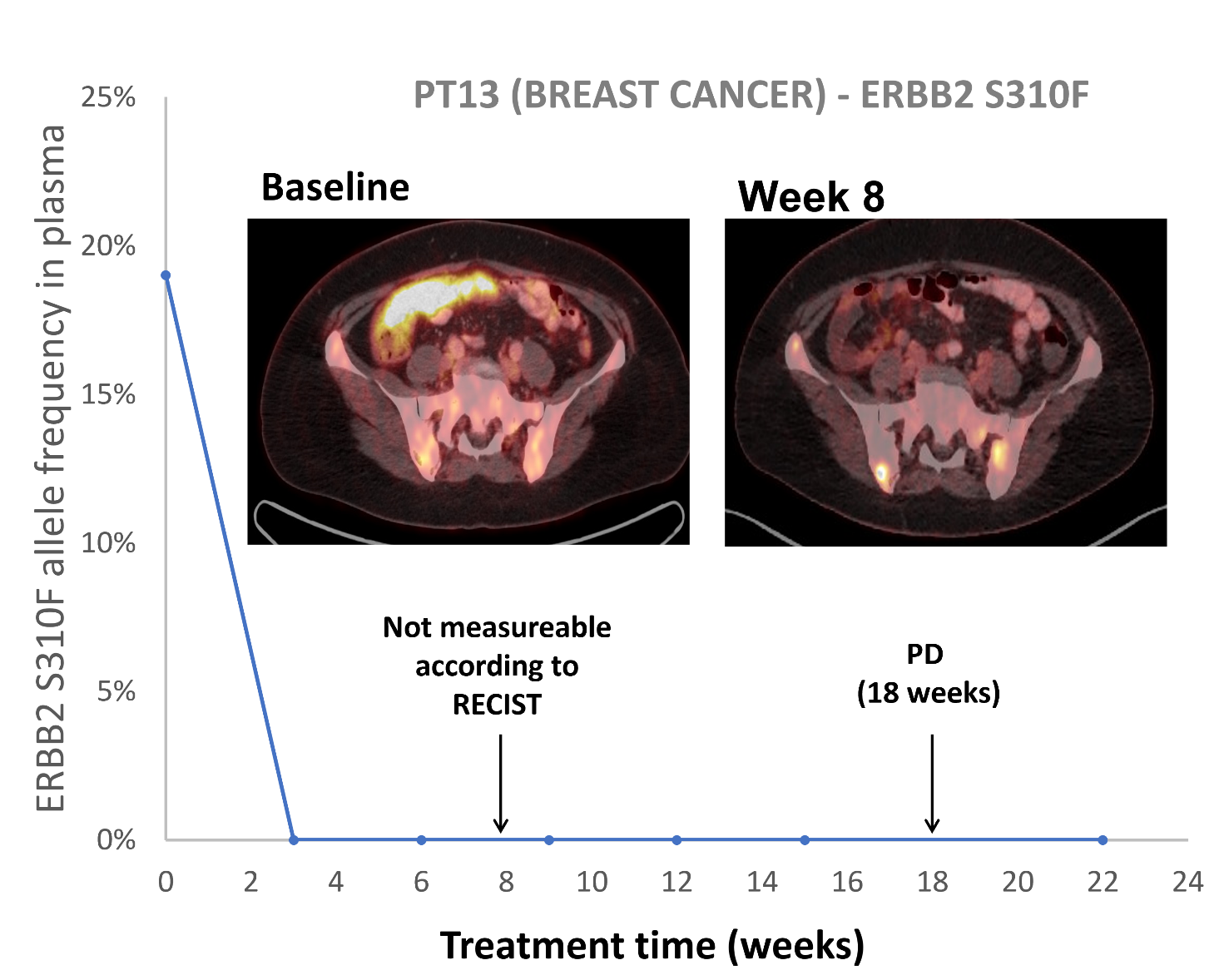

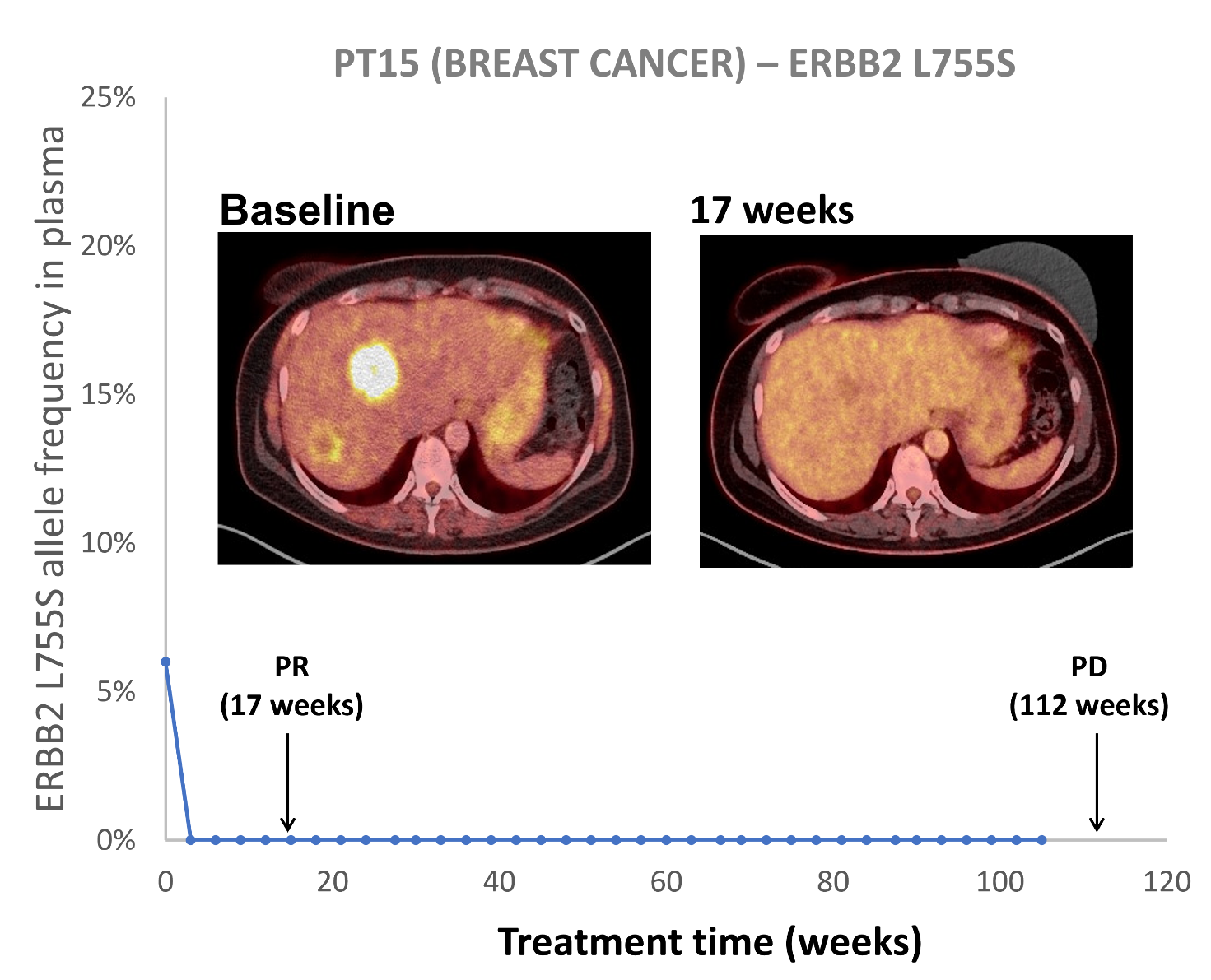

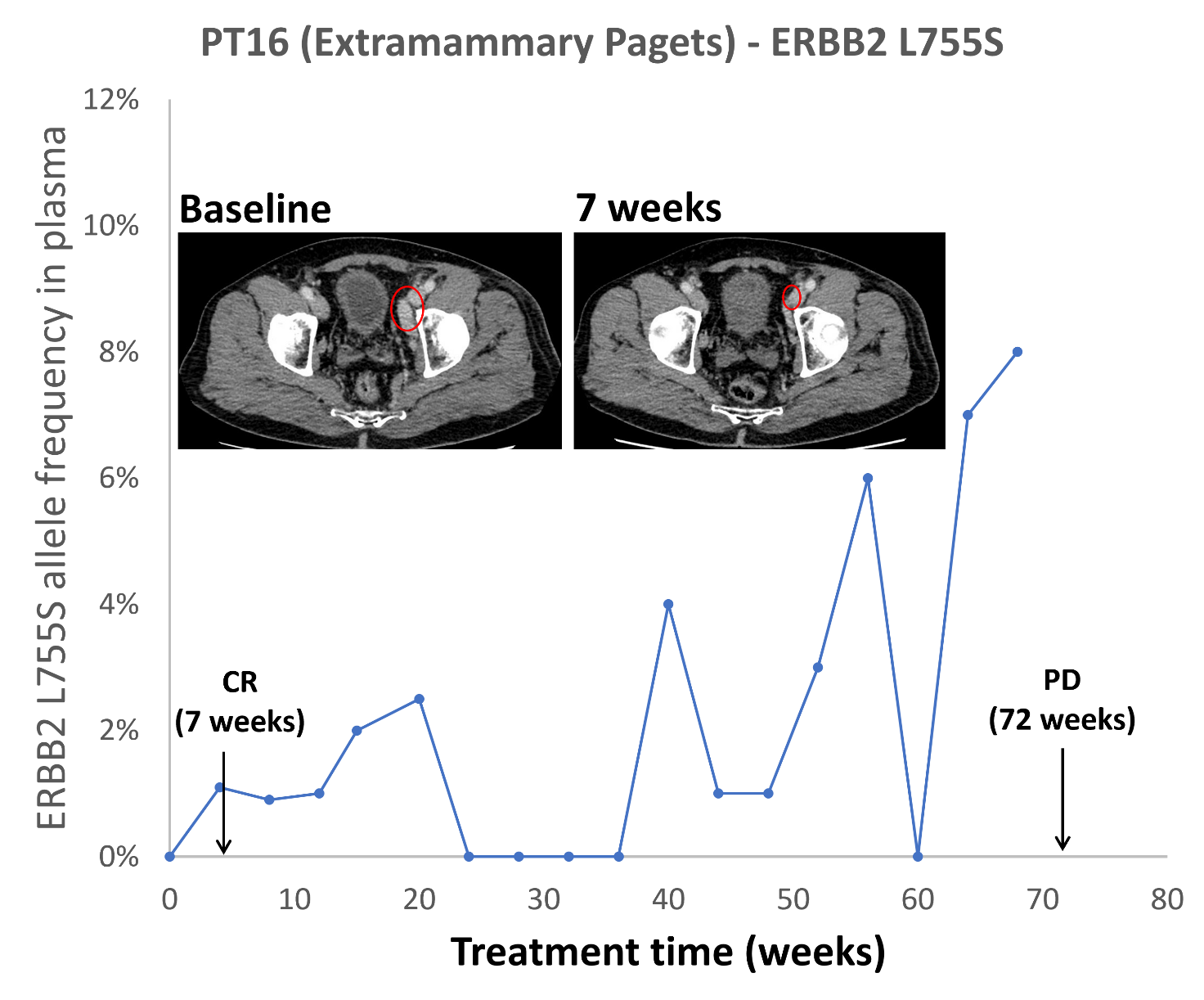

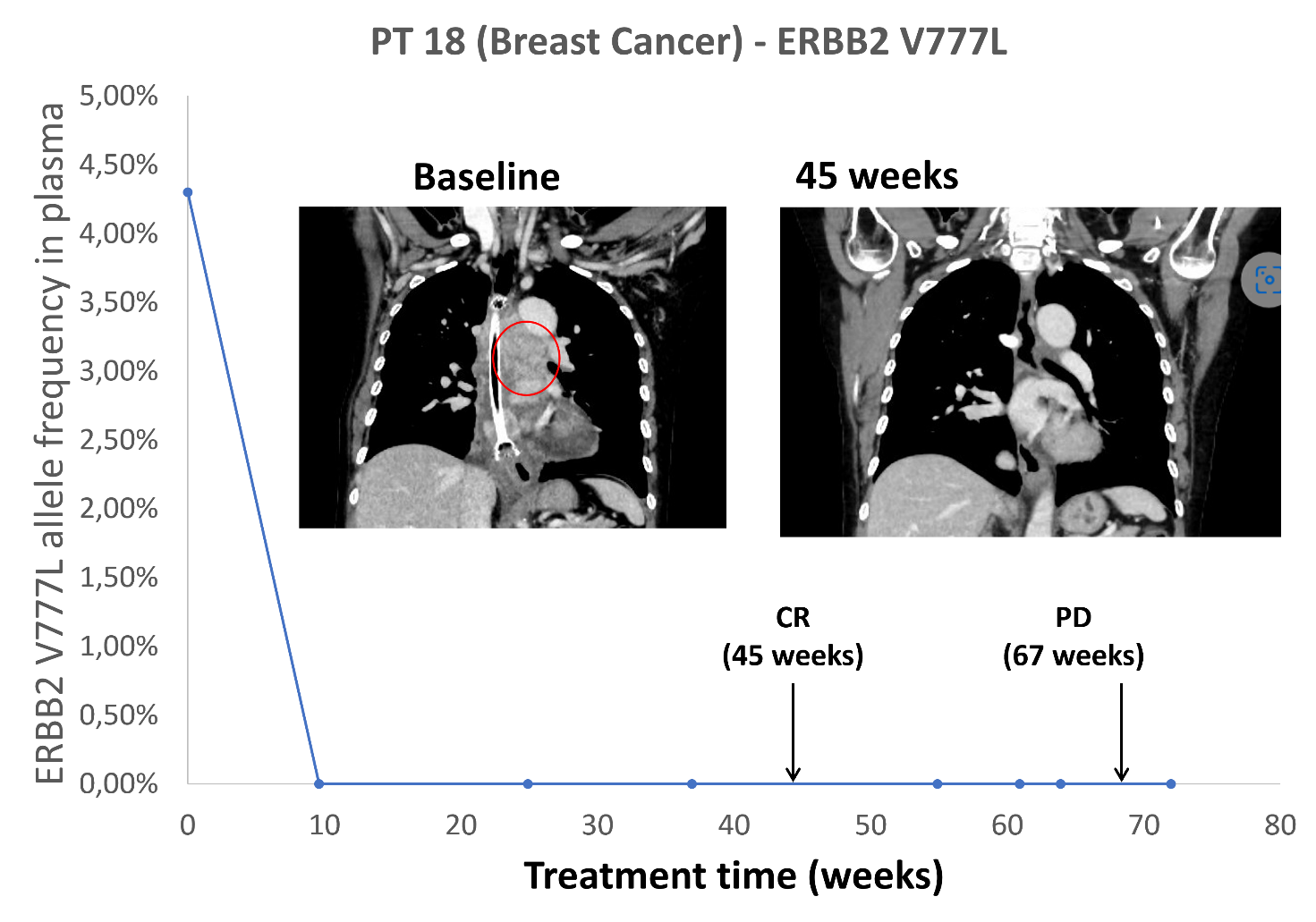


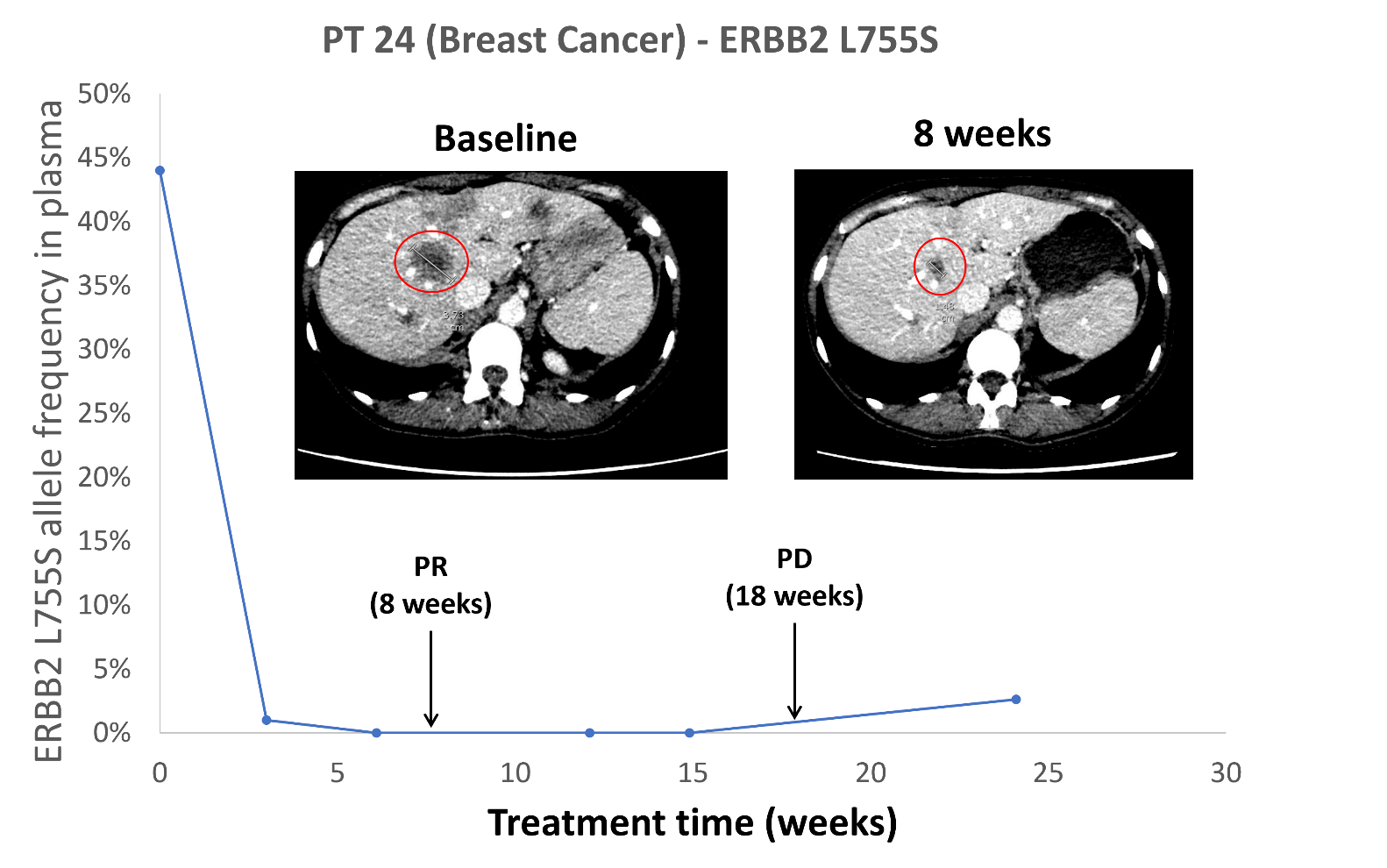

Supplement: Supplementary file 1 — Supplementary Material 1. [file 12885_2025_14599_MOESM1_ESM.docx]
